# Simultaneous recordings from vestibular Type I hair cells and their calyceal afferents in mice

**DOI:** 10.3389/fneur.2024.1434026

**Published:** 2024-08-27

**Authors:** Donatella Contini, Gay R. Holstein, Jonathan J. Art

**Affiliations:** ^1^Anatomy and Cell Biology, College of Medicine, University of Illinois Chicago, Chicago, IL, United States; ^2^Department of Neurology, Icahn School of Medicine at Mount Sinai, New York, NY, United States

**Keywords:** vestibular system, synaptic transmission, potassium conductances, hair cells, calyx

## Abstract

The vestibular hair cell receptors of anamniotes, designated Type II, are presynaptic to bouton endings of vestibular nerve distal neurites. An additional flask-shaped hair cell receptor, Type I, is present in amniotes, and communicates with a chalice-shaped afferent neuritic ending that surrounds the entire hair cell except its apical neck. Since the full repertoire of afferent fiber dynamics and sensitivities observed throughout the vertebrate phyla can be accomplished through Type II hair cell-bouton synapses, the functional contribution(s) of Type I hair cells and their calyces to vestibular performance remains a topic of great interest. The goal of the present study was to investigate electrical coupling between the Type I hair cell and its enveloping calyx in the mouse semicircular canal crista ampullaris. Since there are no gap junctions between these two cells, evidence for electrical communication would necessarily involve other mechanisms. Simultaneous recordings from the two cells of the synaptic pair were used initially to verify the presence of orthodromic quantal synaptic transmission from the hair cell to the calyx, and then to demonstrate bi-directional communication due to the slow accumulation of potassium ions in the synaptic cleft. As a result of this potassium ion accretion, the equilibrium potentials of hair cell conductances facing the synaptic cleft become depolarized to an extent that is adequate for calcium influx into the hair cell, and the calyx inner face becomes depolarized to a level that is near the threshold for spike initiation. Following this, paired recordings were again employed to characterize fast bi-directional electrical coupling between the two cells. In this form of signaling, cleft-facing conductances in both the hair cell and calyx increase, which strengthens their coupling. Because this mechanism relies on the cleft resistance, we refer to it as resistive coupling. We conclude that the same three forms of hair cell-calyceal transmission previously demonstrated in the turtle are present in the mammalian periphery, providing a biophysical basis for the exceptional temporal fidelity of the vestibular system.

## Introduction

Angular and linear accelerations of the head are detected by sensory receptors located in the semicircular canals and otolith organs of the vestibular system (1). The vestibular hair cell (HC) receptors transduce these stimuli into a signaling code that communicates the speed and direction of head movements as well as the position of the head in space (2, 3). Overall, the vestibular end organs have an extremely broad range of sensitivity, and this breadth is conveyed centrally by the vestibular nerve to recipient targets in the brainstem and cerebellum (4). Not surprisingly, the major effector pathways from the vestibular brainstem reflect this diversity: vestibulo-ocular and -collic pathways mediating several of the fastest reflexes in the body, vestibulo-thalamic and -autonomic projections reflecting more moderate dynamics (5) and the lateral vestibulo-spinal tracts engaging both rapid and delayed spinal reflexes (6).

Two types of HCs are present in the vestibular end organs of amniotes (7). Each Type I HC is enveloped everywhere except the apical neck by the calyceal ending of a vestibular nerve afferent. Synaptic contacts between these two cells occur through somato-dendritic ribbon synapses. Type II HCs innervate vestibular afferent processes ending in boutons and, also, contact the outer faces of adjacent calyces; both connections are mediated by ribbon synapses. Recently, the Type I HC-calyx contacts have garnered attention as a possible substrate for a form of extremely fast transmission that has been reported emanating from the striolar regions of the otolith organs and the central regions of the semicircular canal cristae ampullares (8–11). This form of transmission has been suggested by us and others to reflect direct electrical coupling between the closely adjacent HC and calyceal membranes, and to occur in concert with intercellular communication via classic quantal glutamatergic synapses and through the relatively slow accumulation of potassium ions in the interposed cleft (12).

The present study was conducted to identify and characterize the attributes of resistive coupling in the Type I HC-calyceal signaling unit of the mouse semicircular canal crista. The narrowness of the cleft between these two cells creates an electrical access resistance to the surrounding perilymph and, in theory, creates a structure that couples the two elements in a bidirectional, reciprocal pair. During mechanical stimulation of the HC, current flowing out of the basolateral surface of the cell alters the ion concentrations in the cleft. Subsequent depolarization of the pair causes plasmalemmal ion channels to open. This creates a resistive pathway between the pair through which currents from each can flow into the synaptic partner and/or out the cleft into the perilymph. Recent experimental evidence from single- and dual-electrode recordings, as well as pharmacological and theoretical work, supports this concept (8–11, 13–16). Based on paired HC and calyx recordings, we find that the key features of resistive coupling in mice closely resemble those previously described in turtle (8, 12, 14), and are consistent with the notion that fast, bidirectional, voltage-dependent electrical transmission is achieved at this mammalian synapse not through gap junctions, but through the recruitment of ion channels on the basolateral HC and the inner leaflet of the enveloping calyx afferent.

## Materials and methods

### Ethical approval

Experiments used C57BL/6 mouse pups obtained from Institutional wild-type breeding lines with dams obtained from a commercial vendor (Jackson Laboratory). Animals were housed in the AAALAC-accredited UIC Biologic Resources Laboratory. Experimental protocols were approved by the University of Illinois Chicago Institutional Animal Care and Use Committee, following National Institutes of Health guidelines (Animal Welfare Assurance #A3460-01).

### Tissue harvesting and visualization

Tissue was obtained from 225 mice of both sexes (104 females; 121 males), ranging in age from P6 to P21. Mice were anesthetized with isoflurane (17) and decapitated. The head was split sagittally and placed in 4°C artificial perilymph (in mM): 121 NaCl, 5.8 KCl, 1.3 CaCl_2_, 0.9 MgCl_2_, 10 NaHEPES, 2 Pyruvate, 0.7 NaH_2_PO_4_, pH 7.5, 282 mOsm. All reagents were obtained from MilliporeSigma or Fisher Scientific. The brain was entirely removed, save for a small portion of the brainstem that remained attached to the vestibular periphery by the vestibular nerve.

**Figure 1:**
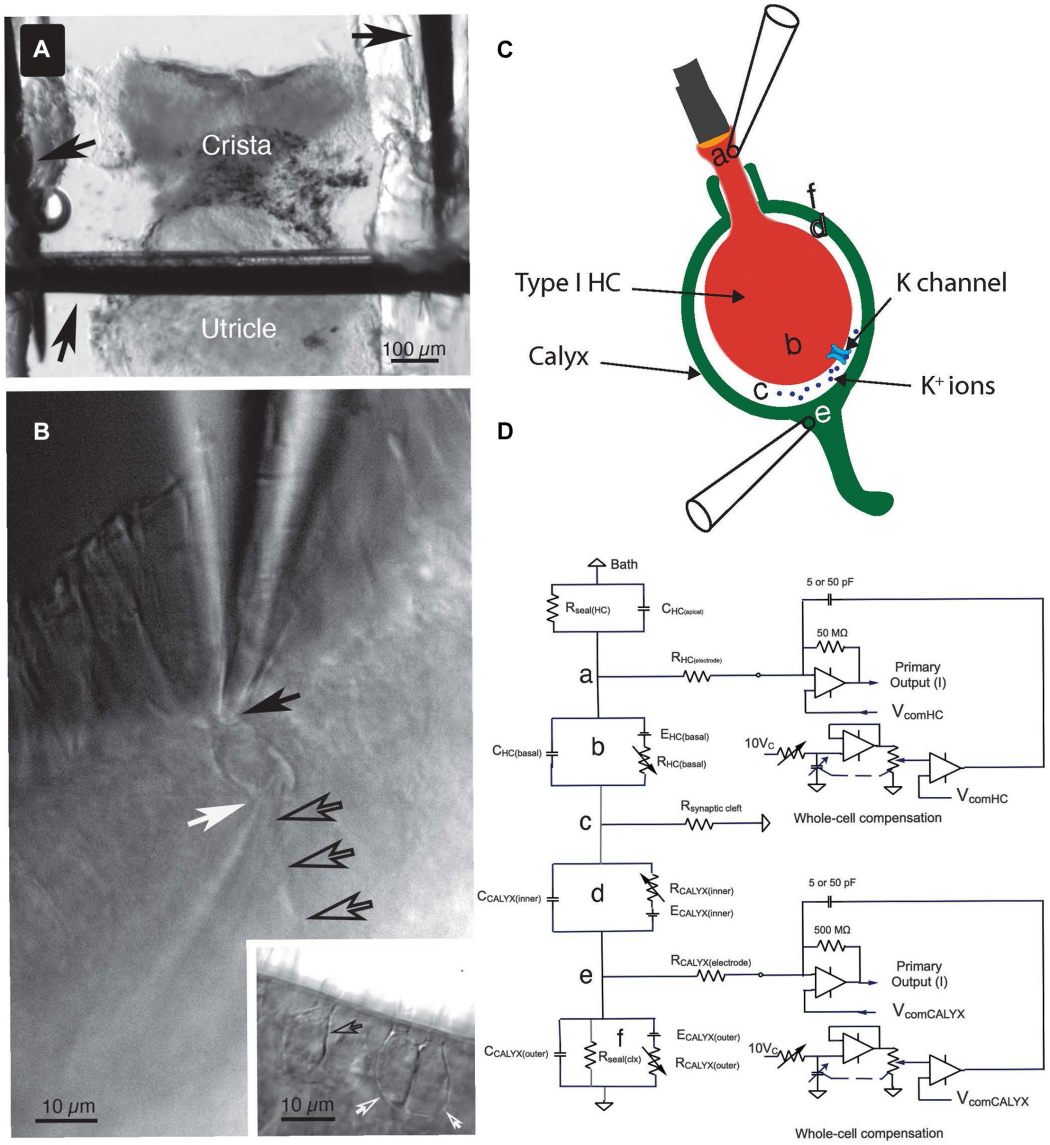
Key features of the experiment setup. **(A)** The crista ampullaris of the anterior semicircular canal, together with the utricle, were secured to the bottom of the recording chamber with minutien pins (black arrows). **(B)** To accomplish the dual electrode recordings, one pipette was positioned at the apex of the Type I HC (solid black arrow) and the other was placed at the base of the calyx (white arrow), near its junction with the parent process (open black arrows). Inset: examples of single (open black arrow) and complex (open white arrows) calyces. **(C)** The anatomical sites of the circuit components (a–f) identified in **(D)** are illustrated schematically. **(D)** Equivalent circuit for the voltage clamp experiments. (a) The seal between the electrode and the Type I HC, (b) circuit representing the Type I HC basolateral membrane, (c) the junction with the synaptic cleft resistance, (d) circuit representing the inner face membrane of the calyx, (e) the seal between the electrode and the calyx outer face membrane, and (f) the circuit representing the outer face membrane of the calyx.

All experiments used the anterior semicircular canal. This canal and the utricle were isolated as a unit. They were transferred in a buffer-filled silanized Pasteur pipette to the 320 μL recording chamber. The canal was immobilized with a 100 μm minutien pin positioned directly above the utricle and inserted into Parafilm strips heat-sealed onto a glass coverslip forming the bottom of the chamber (Figure 1A). The canal vault was opened with tweezers to visualize the crista ampullaris. The epithelium was bisected along the ridge of the crista, perpendicular to the canal axis. The upper side of the epithelium nearest the objective was removed by undercutting the connective tissue to expose the lateral aspect of hair cell-calyx pairs along the ridge. The tissue was then secured laterally with additional pins.

Epithelia were perfused at 1 mL min^−1^ with 24–27°C artificial perilymph supplemented with 0.4 mg mL^−1^ bovine serum albumin (Sigma-Aldrich) and 8 mM dextrose (Fisher Scientific) using a constant-velocity peristaltic pump. For oxygenation, the protein-enriched media flowed through 50 feet of O_2_-permeable silicone tubing immersed in O_2_-saturated buffer prior to entering the chamber.

Electrophysiological experiments were performed using an upright Zeiss Axioskop 2 FS microscope with a 40X, 0.8 NA, 3.6 mm working distance water-immersion objective and 2X Optovar magnification. High-velocity suction was applied to an auxiliary chamber connected to the recording chamber via a 100 μm channel at the coverslip to minimize fluctuations in fluid depth at the objective. Differential interference contrast (DIC) and widefield fluorescence video images were captured with either an iXon_3_ 897, EMCCD camera, QE > 90% (Andor Technology, Belfast, United Kingdom) or a Prime BSI Express CMOS camera (Teledyne Photometrics, Tucson, AZ, United States).

### Electrophysiology

Recordings were obtained from the central and peripheral zones of the epithelia. For dual recordings, one electrode was positioned at the apical neck of the Type I HC, with the second electrode placed at the base of the calyx near its junction with the parent afferent (Figures 1B,C). Highest seal resistances were achieved with borosilicate electrodes (1B150F-4, World Precision Instruments) filled with a mixed KF:KCl intracellular solution (in mM): 118 KF, 12 KCl, 5 K_2_-EGTA, 5.45 K-HEPES, 5 Na_2_ATP, 0.17 Li_2_-GTP, at pH 7.2, to which 50 μM Alexa Fluor 488 or 568 (Thermo Fischer Scientific) was added in order to distinguish the synaptic partners. Electrodes had an average initial series resistance of 5.5 MΩ in the bath.

For pharmacological experiments using intracellular 15 mM 4-Aminopyridinium (4-AP^+^) alone or with 30 mM Tetraethylammonium Chloride (TEA; both from Sigma-Aldrich), the intracellular blocker(s) replaced a chloride-based intracellular solution on an equimolar basis (in mM): 115 KCl, 5.3 MgCl_2_, 5 K_2_EGTA, 0.45 CaCl_2_, 10 KHEPES, 5 creatine phosphate, 5 Na_2_ATP, 0.17 Li_2_-GTP, at pH 7.2. This substitution was done to avoid compromising any potential calcium-activated currents. For pharmacological experiments using 30 mM extracellular TEA in conjunction with intracellular 4-AP^+^, NaCl in the perfusion buffer was replaced on an equimolar basis.

Simultaneous recordings from HC and afferent were made with a MultiClamp 700B Microelectrode Amplifier (RRID:SCR_018455; Molecular Devices, Sunnyvale, CA, United States). Junction potentials were offset prior to effecting a seal. The equivalent electrical circuit of the dual recording topology (Figure 1C) is illustrated schematically in Figure 1D. Series resistance compensation up to 63%, filtered at 4.5–5 kHz, was used to increase the speed of the clamp. As with prior recordings [see (14)], the predictive circuit was not used. The “Whole Cell” circuit was employed in some experiments to cancel transients associated with voltage-command transitions. In these recordings, the cancellation was found to be extremely sensitive to precise adjustment of the fast and slow pipette compensation circuits and often left small residual transients either in phase or inverted with respect to the capacity transients expected for depolarization and hyperpolarization.

The voltage commands illustrated in the figures have been corrected for the instantaneous voltage drops across the electrode and access resistances in all I-V, G-V, and E_Rev_ plots. Voltage commands of 25 ms steps were presented at 200 ms intervals; 500 ms steps were presented every 2 s. In most experiments, whole-cell currents were converted with feedback resistors of 500 MΩ and 50 MΩ for the afferent and HC, respectively. Currents and voltages were low-pass filtered at 10 kHz using 4-pole Bessel filters. All voltage and current signals were digitized at 200 kHz for 25 ms steps and 20 kHz for 500 ms steps. All digitization and voltage commands were accomplished by a Molecular Devices DigiData 1440 digitizer (RRID:SCR_021038). Stimulus protocols and digitization were under software control (Molecular Devices, pClamp 11 (RRID:SCR_011323)). Data were imported using IGOR-compatible routines (NeuroMatic v3_0s) (18). Off-line signal processing, data analysis and figure creation used technical graphing and analysis software (IGOR Pro V9), as previously described (8, 14).

In prior publications, we referred to the total access resistance of the recordings as the sum of the electrode series resistance and that of the synaptic cleft (8, 14). Here we adopt the nomenclature of Armstrong and Gilly (19) and refer to the resistances connected to points (a) and (e) in Figures 1C,D as the series resistance of the HC, R_(HC elec)_, and the afferent, R_(Calyx elec)_, electrodes. In the present study, the access resistance denotes the synaptic cleft resistance, R_(synaptic cleft)_, as shown in Figure 1D(c). Pulses of ±10 mV at a holding potential of −100 mV were used to elicit capacity transients and steady-state (SS) current. This potential is largely depolarized to activation of the HC I_HCN_ (14, 20), but hyperpolarized to activation of the dominant outward I_K(LV)_ (21).

Current amplitudes were estimated by averaging intervals just prior to returning to the holding potential (see Results). The measured current was the sum of the time- and voltage-dependent ionic currents and a linear leak through the seal resistance. To estimate the seal current, a line was fit through the currents at −110 mV, −90 mV, and the point *I* = 0 nA at 0 mV. The current through the seal was subtracted from the total current at each potential to isolate the amplitude of the biologically relevant current. Electrode-HC seals were rarely >1 GΩ; usable data were achieved with seal resistances >100 MΩ.

## Results

**Figure 2:**
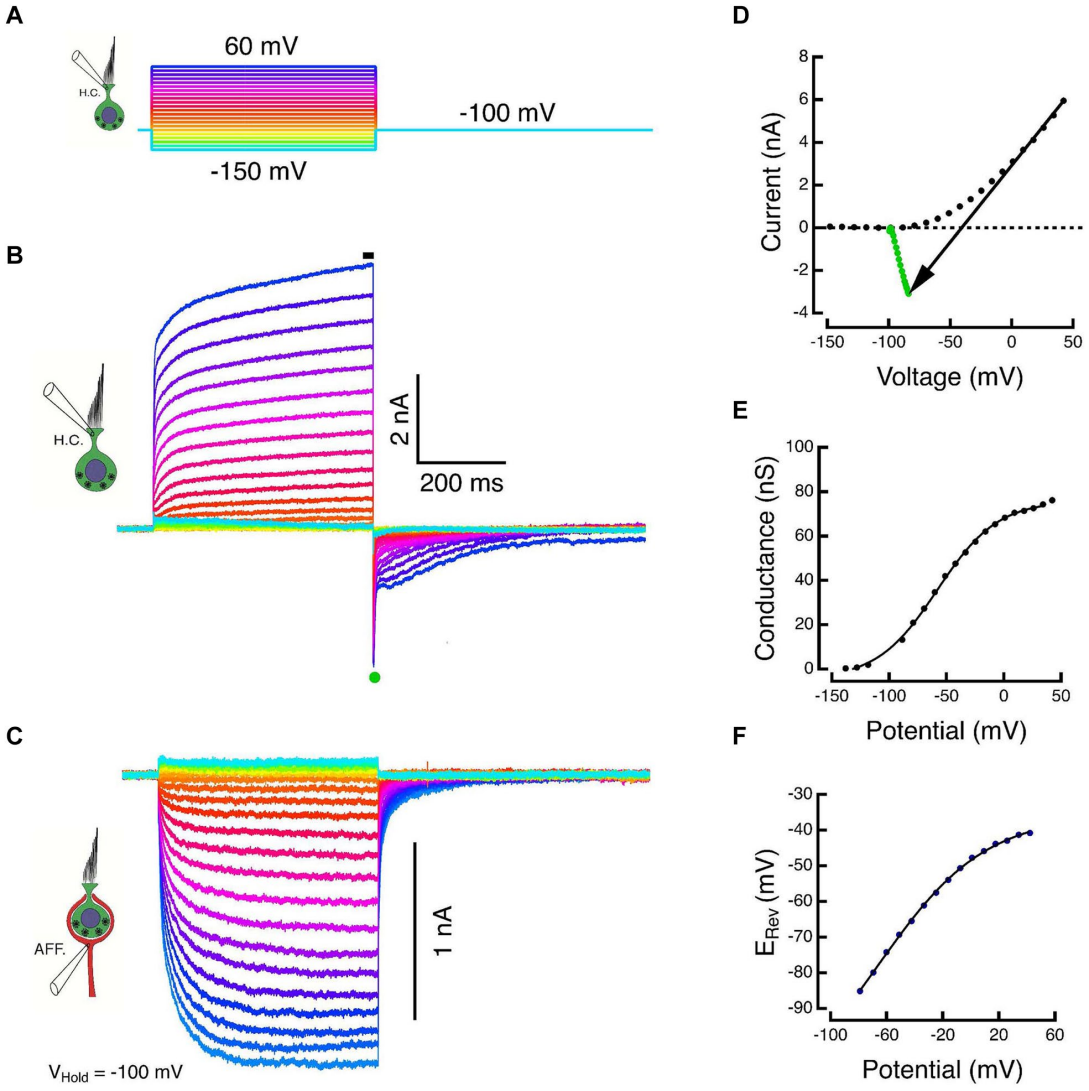
Paired responses of Type I HC and its calyx in voltage clamp. **(A)** Voltage commands to a Type I HC held at −100 mV and stepped between −150 and 60 mV in 10 mV increments. **(B)** Outward HC currents plotted upward. Scaled versions of the capacity transient and the linear leak have been subtracted from each trace. Average of SS current taken at black bar. **(C)** Corresponding inward currents in the associated calyx held at −100 mV, plotted downward. **(D)** The steady-state current during the final 5 ms of the step (black dots) and the average of 500 μs of the peak tail current after HC repolarization to −100 mV (green dots). The slope of the black arrow connecting steady-state and tail currents is the instantaneous conductance during repolarization. The voltage at which the line intersects zero current is an estimate of the reversal potential, E_Rev_, of the net current. **(E)** Chord conductances plotted against corrected membrane potentials were fit with a sigmoid function (see text). **(F)** Shift in the HC reversal potential plotted against the corrected membrane potential during the step.

Orthodromic transmission between HC and calyx was demonstrated by clamping both components within their regions of high impedance where the open probability of membrane conductances is low. A series of hyperpolarizing and depolarizing commands were used to demonstrate the net HC currents and simultaneously to elicit responses in the calyx held at a constant potential. For the experiment in Figure 2, ±10 mV steps, dithered about the holding potential, were used to measure the membrane capacity transient and the ohmic leak through the seal resistance. The capacity transient and leak were scaled by the corrected HC voltages and subtracted from the total current to yield the net ionic current flowing through the HC membrane at each potential.

The HC and calyx were held at command potentials of −100 mV, and the HC was stepped to potentials between −150 and +60 mV for a period of 500 ms (Figure 2A). Increased depolarization produced larger outward currents that were well characterized by the sum of two exponential recovery functions with time constants of 39 ms and 1.1 s at the most depolarized potentials (Figure 2B). The average current during the last 5 ms of the step (black bar in Figure 2B) was plotted against the corrected membrane potential to construct the SS I-V curves (black dots, Figure 2D). Upon repolarization, the outward current reversed as a large transient inward tail comprised of a large rapid phase followed by relaxation through a multicomponent tail. The tail reversal suggests that the command potential of −100 mV is below the equilibrium potential of the major current carrier at the transition. The average current during the 500 μs interval at the peak of the tail (green dot, Figure 2B) was plotted against the corrected membrane potential to construct the instantaneous (INST) I–V (green dots, Figure 2D).

During the HC voltage-command steps, the response in the calyx displays an increase in the inward SS amplitude that is proportional to the increased HC voltage command and corresponding HC outward current (Figure 2C). The HC kinetics are faster and more step-like than the corresponding inward currents in the calyx. The calyx response is consistent with a leaky integration of the components released from the HC. Despite being held nominally at a constant potential, there is an instantaneous decrease in the inward calyx current at the conclusion of the HC step, after which it too relaxes to baseline with a multi-component tail current. This remarkable response was also observed in our prior experiments in the turtle (14). The instantaneous decrease in inward calyx current is a demonstration of one of the fast components of bidirectional HC-calyx coupling due to the synaptic cleft resistance. This is examined in more detail in the last set of experiments described below.

At the HC transition from a step to the holding potential, neither the HC and calyx conductances, nor the synaptic cleft ion or transmitter concentrations can change instantaneously. As a result, the chord between the SS current (black dots, Figure 2D) and the INST current (green dots, Figure 2D) is ohmic. The chord’s slope is the conductance achieved at the end of the preceding pulse and its intersection with the *I* = 0 nA axis is the corresponding equilibrium potential. The conductance increases monotonically to reach a G_max_ = 80.5 nS, and when plotted against the corrected membrane potential, is well fit by the sigmoid function *base* $+\left(\max\;/\;\left(1+\exp\left(\left(\mathrm{xhalf}-\;x\right)\;/\;\mathrm{rate}\right)\right)\right)$ ; with corresponding parameters: −4.5 nS, 80.5 nS, −58 mV, 26.4 mV (Figure 2E). However, the effect on the equilibrium potential is strikingly different from the expected result of recording isolated or solitary cells. In the latter recordings, the external and internal ion concentrations are expected to remain constant since the external bath is effectively infinite and the internal concentrations are determined by the pipette solution. Thus, for solitary cells, the intersection of the SS-INST chord with the zero-current axis would remain fixed. For a HC within the extended embrace of the calyx however, increased HC depolarization results in increased current flow from its basolateral membrane into the synaptic cleft, and the reversal potential depolarizes in a manner consistent with an increase in the relevant ion(s) in the synaptic cleft. The elevation of the reversal potential (Figure 2F) plotted against the corrected membrane potential is also monotonic and can be fit by a sigmoid with parameters: −120 mV, 84.4 mV, −66.1 mV, 38.3 mV.

This result replicates our previous findings in turtle (8, 14). Since prior single-electrode experiments indicated that [K^+^]_cleft_ increased with depolarization (13, 16) while glutamate accumulation exerted relatively modest effects (22), we hypothesized that the major postsynaptic effect on the calyx would be due to basolateral HC potassium currents that elevated [K^+^]_cleft_ and altered the driving force on the cleft-facing calyceal conductances. If so, selective pharmacological blockade of the HC potassium conductances should block the major HC current and the subsequent inward calyx current.

### Pharmacological blockade of HC potassium conductances

Superfusion of blockers in the bath solution could affect conductances in multiple HCs and calyces, thereby confounding our results. To circumvent this, in most experiments we employed an intracellular block of the HC potassium conductances, which allowed us to isolate the effect due to permeation through the basolateral conductances of one individual HC. Without a reliable means of internal perfusion of the HC pipette, this technique precluded characterization of dose–response curves in a single synaptic pair. Our preliminary conclusions are drawn from population studies using multiple pairs with intracellular blocker doses that spanned concentrations over multiple orders of magnitude. In two experiments, intracellular 4-AP^+^ block was combined with extracellular TEA perfusion.

**Figure 3:**
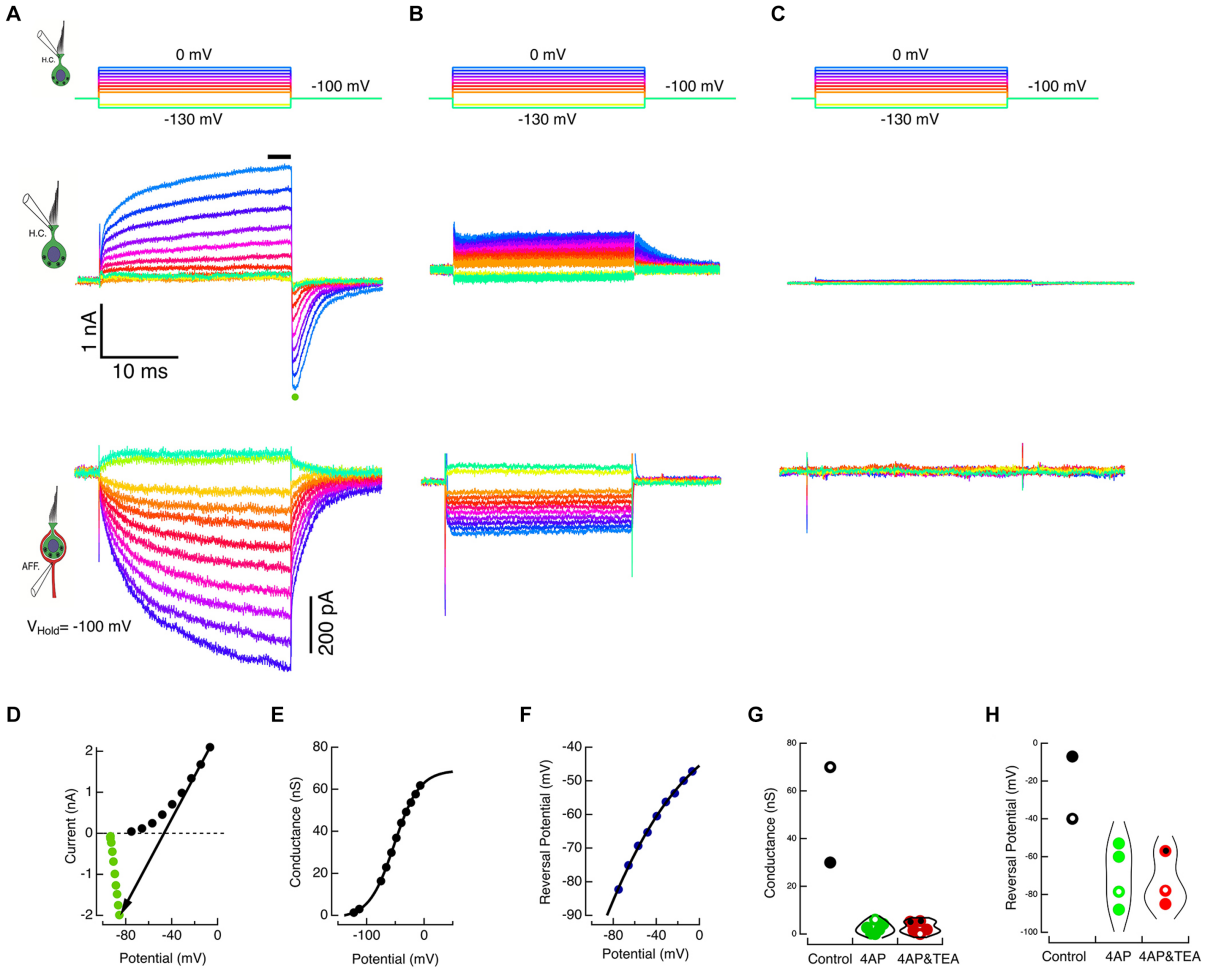
Block of HC potassium permeation decreases K^+^ accumulation in the cleft and associated inward currents in the calyx. **(A)** The HC and calyx of Figure 2 were held at −100 mV. The HC was stepped to command potentials of 25 ms shown in the top panel. Hyperpolarizations turned off the standing current at −100 mV, and depolarizations elicited outward currents in the HC (middle panel) and corresponding inward currents in the afferent (lower panel). **(B)** In a second cell, when 15 mM 4-AP^+^ was substituted in the HC pipette solution, the amplitude of the HC currents was reduced (middle panel) and the associated inward currents in the calyx decreased (lower panel). **(C)** In a third cell, the inclusion of 30 mM TEA and 15 mM 4-AP^+^ in the intracellular solution effectively abolished the HC net current (middle panel) and associated inward currents in the calyx (lower panel). **(D)** The steady-state current at the last 3 ms of the step (black circles) and the average peak tail current for 500 μs (green circles) after repolarization to −100 mV for the HC currents in A are plotted against the corrected membrane voltages. The black arrow connects corresponding steady-state and tail currents, representing the conductance at repolarization to −100 mV. The reversal potential is identified by the point where the arrow intersects the zero current axis. **(E)** Chord conductances are plotted against the corrected voltage potential with conductances fitted with a sigmoid function, $\mathrm{base}+\left(\max/\left(1+\exp\left(\left(\mathrm{xhalf}-x\right)/\mathrm{rate}\right)\right)\right)$ ; with parameters: −1.26 nS, 70.3 nS, −51.8 mV, 21.8 mV. **(F)** Reversal potentials are plotted against the corrected membrane potentials fitted with a sigmoid with parameters: −235.4 mV, 206 mV, −133 mV, 54.4 mV. At the maximum rate, the reversal potential shifted 38.3 mV for each 10 mV of depolarization. **(G)** Conductances are shown for individual cells (black dots), 4 AP^+^ (green dots), and 4 AP^+^ with TEA (red dots). Open circles correspond to cells illustrated in A, B, and C. Symbols with central black dots indicate where external TEA was used with internal 4-AP^+^. **(H)** Reversal potentials for different individual cells are plotted for control (black dots), 4 AP^+^ (green dots), and 4 AP^+^ with TEA (red dots). Open circles correspond to cells illustrated in A, B, and C.

To increase the probability of data capture across the full range, the duration of the HC voltage commands was decreased to 25 ms. With durations only 5% of those used in the experiment of Figure 2, the maximum conductance, G_max_, and the shift in the reversal potential, E_rev_, were less, as expected, given the kinetics of the HC currents. Such an experiment for the cell in Figure 2 is shown in Figure 3A for direct comparison. The HC commands ranged from −130 to 0 mV in 10 mV steps, Figure 3A, top panel. Capacity transients and seal leak currents were subtracted as before, and the net ionic currents are shown in Figure 3A, middle panel. Current flow through the recording electrode depolarized the holding potential to −93.4 mV. The corresponding calyx currents are shown in the bottom panel of Figure 3A. As expected, HC hyperpolarization resulted in a net outward current in the calyx while depolarization elicited proportional inward currents.

The average HC current during the last 3 ms of the step response (black bar, Figure 3A, middle panel) was plotted against the corrected membrane potential to construct the SS I–V curve (black dots, Figure 3D). Upon HC repolarization to the holding potential, the peak inward current (green dot, Figure 3A, middle panel) was plotted against the corrected membrane potential to construct the INST I–V curve (green dots, Figure 3D). The chord connecting corresponding points in the SS and INST I–V curves was used to estimate the conductance (Figure 3E) and the reversal potential (Figure 3F) of the net current at each potential. The conductance was fit with a sigmoid with parameters: −1.26 nS, 70.3nS, −51.8 mV, 21.8 mV. Similarly, the reversal potentials could be fit with sigmoid with parameters: −235.4 mV, 206 mV, −133 mV, 54.4 mV.

In these experiments, we exploited the fact that at pH 7.2, 4-AP in the internal solution converts to 4-AP^+^, and the pyridinium form is retained within the cell. Using HC commands between −130 and 0 mV delivered in 10 mV steps (Figure 3B, top), the response of a second cell filled with 15 mM 4-AP^+^ substituted on an equimolar basis for K^+^ is shown in Figure 3B, middle panel. In the presence of the blocker, net current flow of 1.6 nA through the residual electrode resistance of 28 MΩ resulted in depolarization of the holding potential to −53 mV, and the resulting tail currents upon repolarization remained outward. This suggests that the reduced current was insufficient to raise [K^+^]_cleft_ to concentrations sufficient to create an equilibrium potential depolarized to the corrected holding potential. In the calyx, there is a commensurate reduction in the corresponding inward currents evoked by HC depolarization (Figure 3B, bottom). In a third cell, when 15 mM 4-AP^+^ was augmented with 30 mM intracellular TEA, these small HC currents were reduced still further, and the induced calyx current became vanishingly small (Figure 3C). The average maximum conductances for the two controls 50.0 nS 4-AP^+^ block (2.48 nS, SD = 2.33 nS, *N* = 6), and with 4-AP^+^ combined with intra- or extracellular TEA block (2.79 nS, SD = 2.39 nS, *N* = 5) are shown in Figure 3G. Corresponding reversal potentials for control (−23.5 mV), 4-AP^+^ block (−69.8 mV, SD = 16.19, *N* = 4), and 4-AP^+^ plus TEA block (−73.3 mV, SD = 14.6 mV, *N* = 3) are shown in Figure 3H. The reduction in the induced inward calyx current correlated with these blockades of HC potassium permeation, leading to the suggestion that the elevation in [K^+^]_cleft_ is a major causal mechanism depolarizing E_K_ and generating inward currents in the calyx afferent. This was tested in the next set of experiments, in which K^+^ was directly applied to the afferent.

### Direct ionophoresis of K^+^ onto the calyx

Type I HCs in the mouse are smaller than those in the turtle, which makes the dual-recording configuration more challenging. Moreover, to preserve normal physiology, we did not use low calcium or enzyme solutions (23). Such strategies are typically employed to improve electrode seals by removing peripheral or lipid-anchored membrane proteins, and by inhibiting cupula and tectorial membrane synthesis (23). Consequently, seal resistances in the present study were relatively low, and rupturing the membrane within the electrode to achieve the whole-cell configuration often resulted in partial or complete loss of seal and HC or calyx integrity.

Though recordings after seal failure bore a superficial resemblance to physiologically relevant recordings, they differed in features that were diagnostic. Synaptic pairs for which the HC seal was lost, but a whole-cell recording from the calyx was achieved, were used to investigate the effects of K^+^ ionophoresis onto the afferent, in many respects complementary to the prior potassium block experiments of Figure 3.

**Figure 4:**
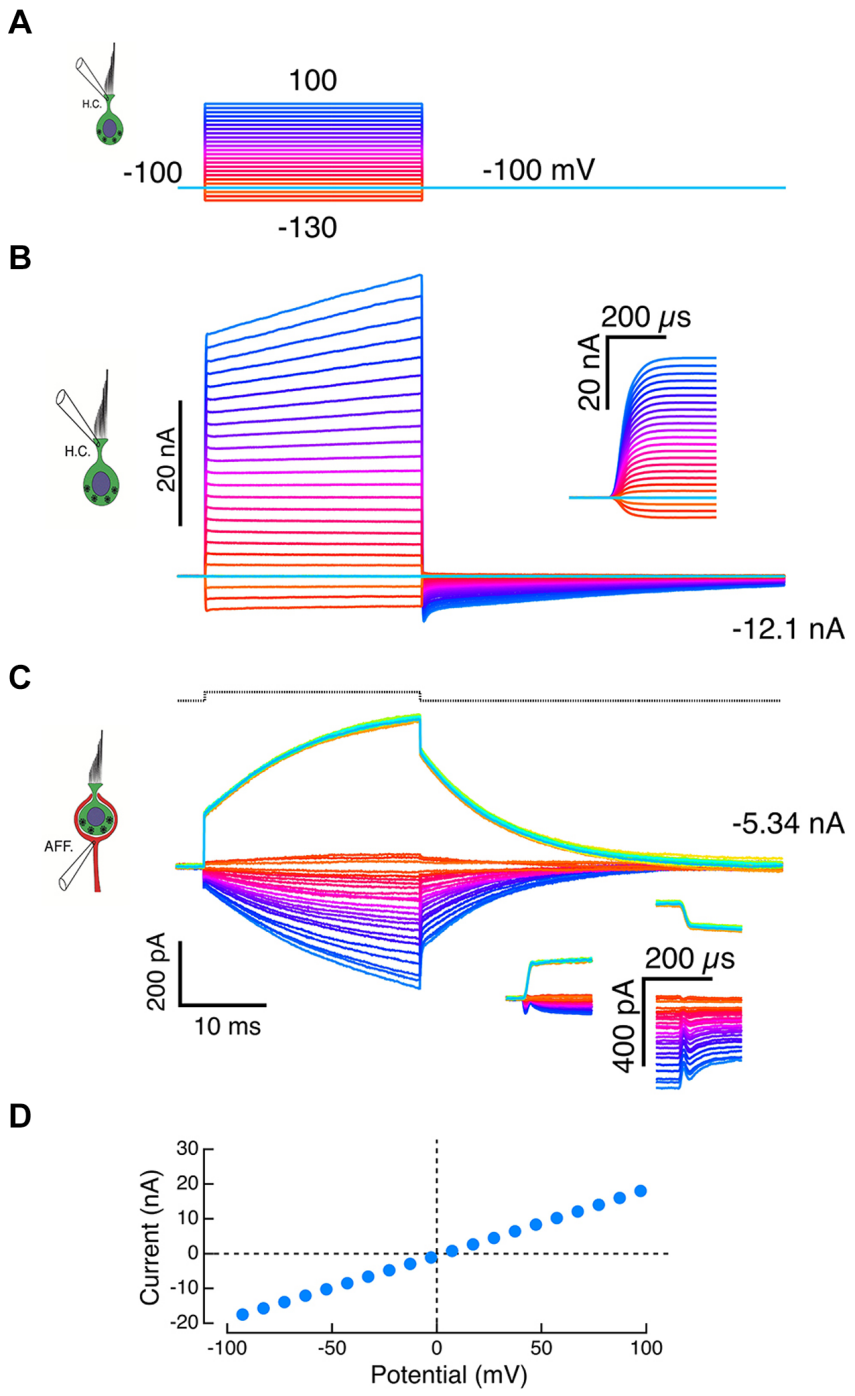
Failure of HC-electrode seal reveals effects of local potassium perfusion on the calyx. **(A)** Voltage commands to the HC pipette. **(B)** A standing inward current of −12.1 nA through the HC electrode was recorded at the holding potential of −100 mV, suggesting a loss of electrode seal to the HC and a corresponding resistance of 8.3 MΩ to the bath. Typically, depolarizations of the pipette generated large instantaneous outward currents (inset: expansion of the first 300 μs) followed by slower additional increases. **(C)** Inward calyx currents of increasing amplitude were recorded in response to larger outward currents recorded from the HC electrode in panel B. These increasing inward currents are consistent with voltage-dependent ionophoresis of K^+^ onto the calyx. Depolarization of the calyx by 10 mV in voltage clamp (black dashed trace at top) evoked calyx currents (green traces) that had instantaneous jumps (insets) and exponential time constants matching those seen with HC-electrode ionophoresis. The slow current increases during the step were well fit by exponential recovery functions. In the calyx voltage clamp experiments, the increased current during the step is likely to reflect an inability to space clamp the parent axon and the recruitment of conductances more distal from the electrode over the duration of the pulse. **(D)** In experiments with compromised HC seals, the I–V curves of the HC recording electrode were linear and intersected the origin when corrected for pipette offset.

After the loss of HC seal, the extent of HC remnants within the calyx were unknown. Given the restricted topology, the observed effects might be the result of changes on either the inner or outer face, or a combination of both. In many cases, depolarizing commands to the HC electrode (Figure 4A) resulted in HC currents that superficially resembled successful recordings but differed in several essential features. At the holding potential of −100 mV, the HC recordings had large inward currents (Figure 4B). When corrected for the pipette offset, the I-V curve passed through the 0,0 point with a slope of 100 nS, corresponding to a 10 MΩ electrode resistance (Figure 4D). Dithering ±10 mV about the holding potential never elicited the expected capacity transients, and the recorded outward currents were limited by the recording bandwidth (Figure 4B and inset). Moreover, at the largest command voltages, the initial rapid rise was followed by a slower additional increase in outward current.

Similarly, inward currents in the calyx were associated with the large HC electrode outward currents (Figure 4C) and resembled those of intact synaptic pair recordings (Figures 2 and 3). However, these calyx currents also differed in important ways from physiologically relevant recordings. Step commands to the HC electrode elicited rapid transitions with intervening exponential increases in the calyx current (Figure 4C, downward currents). The time constants of these currents were within 10% of the time constants of a calyceal response to small step depolarizations (Figure 4C, upward current), a result consistent with sudden changes in [K^+^]_o_ at the beginning and end of the command. The proportional increases in induced current fit with higher [K^+^]_o_ associated with larger quantities of K^+^ expressed at larger command voltages. Since 1 nA is equivalent to 10^9^ ions sec^−1^ from the electrode, we expect that large 30–40 nA currents would significantly elevate [K^+^]_o_. To the extent that this represents elevation of [K^+^] on the inner face, these experiments comport with prior intracellular pharmacological block of the HC potassium conductances. Since 4-AP^+^ is membrane impermeant, it will not block the afferent potassium channels because it cannot reach the high-affinity internal blocking site.

It is important to remember that two components of these calyx recordings are likely artifacts which resemble true physiological responses and might be confused with normal physiology. They differ in that the rapid transitions at the onset and offset of the step are symmetric, and the time constant of increased current during the command step matches the time constant of the calyx in response to +10 mV depolarizing voltage commands. The true physiology of fast and slow bidirectional transmission can only be assessed in preparations where dual recordings result in non-linear voltage-sensitive activation of both HC and calyx conductances.

### Bidirectional fast transmission in HC and calyx

Our dual-electrode recordings in mice demonstrate that slow potassium accumulation in the synaptic cleft creates coupling between the HC and calyx due to shifts in the potassium equilibrium potential for cleft-facing HC and afferent conductances. This is consistent with prior single-electrode recordings in mice (13, 16, 24) and dual-electrode recordings in turtle (8, 14). Dual recordings in turtle were also used to demonstrate a second, and perhaps evolutionarily more important, fast coupling between the two cells of the synaptic pair. The last set of experiments sought to investigate this fast coupling in the mouse crista.

**Figure 5:**
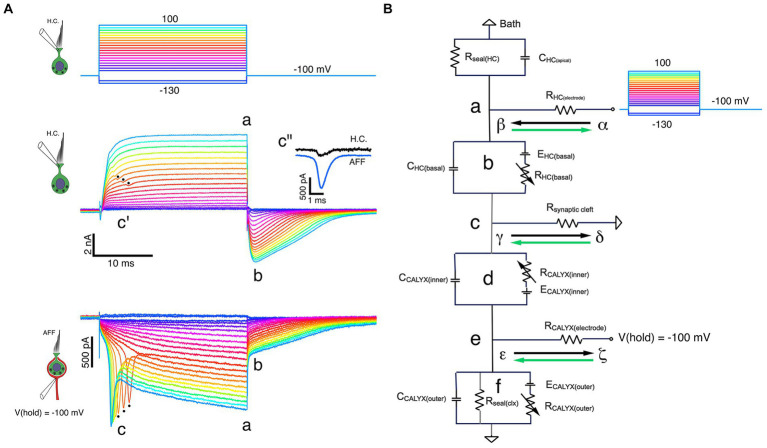
Fast and slow bidirectional coupling between HC and calyx afferent demonstrated in dual recordings. **(A)** Voltage commands to the HC held at −100 mV and stepped between −130 mV and 100 mV in 10 mV steps (top panel). Depolarizations resulted in proportional outward currents in the HC (middle panel), followed by inward tail currents upon HC repolarization. Scaled versions of capacity transients and steady-state leak have been subtracted. Inward calyx afferent currents (bottom panel) are proportional to the size of the corresponding HC currents. For the nine largest induced currents, action currents from the more proximal (re: CNS) spike initiation zone were back-propagated into the calyx at c. These were correlated with small, phase-locked decreases in the outward current visible in the corresponding outward HC currents (middle panel, c’). The afferent action current and deviation in the HC outward current are phase locked with negligible delay (middle panel inset, c”). At the voltage command termination, there were rapid inversions of the HC current from outward to inward, from ‘a’ to ‘b’. With the calyx held at a constant −100 mV, there was a contemporaneous decrease in the inward current (bottom panel, ‘a’ to ‘b’) before returning to baseline. **(B)** Equivalent circuit of dual-recording illuminates features of fast and slow coupling between HC and calyx. At the initiation of a depolarizing command to the HC, currents flowing from α to β (black arrow) through R_HC (electrode)_ to point ‘b’. Outward current through R_HC (basal)_ flows from γ to δ (black arrow) from point ‘c’ within the cleft, along R_synaptic cleft_ to ground, resulting in depolarization of point ‘c’ with respect to ground. During the pulse, K^+^ flowing from the HC increases [K^+^]_cleft_, shifting both E_HC(basal)_ and E_CALYX(inner)_ to less negative potentials. Upon HC repolarization, inward current flows from δ to γ (green arrow) along R_synaptic cleft_ to point ‘c’, then into the HC (‘b’) and out through R_HC (electrode)_ from β to α (green arrow). This current flow hyperpolarizes point ‘c’ with respect to ground.

With the calyx held at −100 mV, increased depolarization of the HC brings it to potentials where sufficient calyx inward current triggers action potentials from a spike initiation zone more proximal to the central nervous system. Since the calyx cannot be space-clamped using a single electrode, the associated action currents back-propagate into the calyx. Three such currents with the longest latency are shown in the bottom panel of Figure 5A (black dots at c). The dot at the peak of each action current corresponds to the dip in the outward current (Figure 5A, middle panel c’), suggesting that there is a decrease in the driving force on the channels that are open at those HC potentials. To compare the timing of the two traces, the HC current step response was fit with a double-exponential recovery function, and the afferent response fit with a double-exponential function, and these fits were subtracted from corresponding currents to demonstrate the phase locking of the two (Figure 5A, middle panel c”).

The rapid coupling is also evident at the termination of each step, when outward HC currents at ‘a’ rapidly transition to inward currents at ‘b’ (Figure 5A, middle panel). A sudden decrease in the inward calyx current (from ‘a’ to ‘b’) followed by a slowly relaxing tail current is synchronized with the HC current reversal (Figure 5A, lower panel). This occurs despite holding the calyx nominally at a constant command of −100 mV. These data indicate that there is a sudden decrease in the driving force across the afferent conductances.

As a starting point, we assume that the driving force across the outer face of the calyx remains unchanged, and that any decrease in the inward current is associated with a change in the driving force across the inner face calyx conductances, and therefore a change of the potential within the cleft. In Figure 5B, a schematic of the recording configuration, we assume all components can be lumped, since as yet a detailed map of conductance expression is unavailable. When depolarizing commands are delivered to a HC held at −100 mV, outward current through the basolateral HC membrane flows from α to β down the electrode series resistance, into the HC at ‘b’ where it flows through a battery equivalent to the instantaneous equilibrium potential, across the membrane conductance, and finally down the synaptic cleft access resistance ‘c’ from γ to δ to ground. Flow from γ to δ results in γ being positive relative to ground. With respect to the calyx positivity, ‘c’ results in further hyperpolarization of inner face channels. As current continues to flow, the [K^+^]_cleft_ increases, changing the equilibrium potentials for channels on the basolateral HC membrane and those on the inner face of the calyx. We assume any K^+^ permeation through outer face calyx channels is free to diffuse away, and as a result the equilibrium potential for outer face channels remains unchanged.

Upon repolarization, the HC current reverses and current into the HC flows from δ to γ in the cleft, through the HC membrane conductance from ‘c’ to ‘b’ and into the electrometer at ‘a’ from β to α. The flow in the cleft from δ to γ means that ‘c’ is now instantaneously hyperpolarized. This corresponds to a depolarization of the channels on the inner face of the calyx, bringing them closer to the equilibrium potential. This decreases the driving force on the channels, even though the command potential to the calyx has not changed. The main effect at ‘e’ will be on the inner face channels, although the change in current flow will also change the potential at ‘e’ due to current flow across the calyx electrode.

In paired recordings such as these, the HC is largely surrounded by the calyx and the homogeneous environment of the synaptic cleft. The calyx, however, has two distinct response components: one from the inner face where the [K^+^]_cleft_ changes dynamically, and the other at the outer face where [K^+^]_o_ is relatively constant. A single electrode recording the afferent current is unable to reveal what percentage is flowing through each of these faces. Potential gradients and the resulting equivalent batteries are usually generated by slow mechanisms such as pumps and ion exchangers, or in the case of the cleft, by current flow through membrane conductances. These equivalent batteries are created by differences in ion concentration where the electrical and osmotic forces balance. HC basolateral and calyx inner face conductances experience changing batteries as the current from either the HC or the calyx flows in and out of the synaptic cleft. The fast, instantaneous changes in current, by contrast, could be due either to capacitive coupling, or to the balance of currents that flow across the HC, calyx, and cleft conductances, resulting in changes in driving force across open conductances as described by Ohm’s and Kirchhoff’s laws.

## Discussion

Except for the proto-hemicalyx in the fish crista, in which the HC membrane protrudes into the afferent in a “glove-and-finger” arrangement (25), boutons are the sole and ubiquitous afferent endings across all anamniotes (26). The specialized calyx and associated Type I HC emerge with the evolution of amniotes, and a hyperexpression of the calyx ending is seen in the complex calyces of birds, in which 2–15 HCs may innervate a single afferent (27). It is not yet known whether the channel expression and response dynamics of individual HCs within a complex calyx such as this are homogeneous or exploit differential channel expression to extract complex features of a stimulus. Initial speculation based on ultrastructure posited two distinct regions of calyceal communication: one involved in vesicle-based quantal synaptic transmission and a second area of close membrane apposition potentially utilizing gap junction-mediated electrical transmission (28). However, subsequent analysis failed to identify the distinctive morphological features of gap junctions in the contacts between HCs and calyces, leading to the conclusion that typical electrical coupling does not exist between these cells (29). The gap junctions that do exist in the vestibular periphery are present between adjacent supporting cells, where they are hypothesized to maintain ionic and metabolic homeostasis (30).

We have previously reviewed and compared the response dynamics and regularity of discharge in bouton, calyx, and dimorphic units consisting of a combination of bouton and calyx endings across the dendritic arbor of a single afferent (12). It has been established that the full range in afferent discharge regularity, as well as the complete repertoire of acceleration, velocity, and displacement-sensitive units can be achieved in species that rely solely on bouton afferents (31). Moreover, the span of regularity and response properties in calyx and dimorphic units are correlated primarily with terminal location within the epithelium, and not with the existence of calyces *per se*. Calyx afferents with irregular discharge and phasic dynamics are often located centrally, near the torus in species that have one, and those with more regular afferent discharge and tonic responses are located near the planum. Taken together, these observations raise the question of the functional significance of the HC-calyx configuration for the vestibular system.

The present study examined evidence for fast electrical coupling between the Type I HC and its enveloping calyx in the absence of gap junctions using a mouse model of mammalian vestibular signaling. Employing simultaneous recordings from the two cells of the synaptic pair, we initially verified orthodromic transmission from the HC to the calyx, indicative of classic synaptic neurotransmission. The major HC outward current, and the subsequent inward current in the calyx, were essentially extinguished by intracellular pharmacological blockade of the HC potassium conductances. This reduction in the induced inward calyx current correlated with the HC potassium permeation block, suggesting that elevated [K^+^]_cleft_ is responsible for the inward currents normally observed in the calyx, comprising a second and slower form of intercellular communication. Lastly, we pursued fast electrical coupling between the HC and calyx. Through a series of paired simultaneous recordings, we were able to identify bi-directional resistive coupling, and provide an equivalent circuit model to explain this form of ultrafast intercellular communication. We conclude that the same three forms of HC-calyceal transmission previously demonstrated in the turtle are present in the mammalian periphery, providing a biophysical basis for the enormous temporal range of vestibular sensitivity.

### Slow accumulation at the synapse between HC and calyx afferent

Although potassium accumulation may be the dominant determinant, the slow component of transmission may consist of a combination of ions (13, 16, 32) and transmitter accumulation (22, 33) with subsequent diffusion or reuptake from the cleft. The HC SS and INST I-V curves were used to estimate the voltage dependence of the HC membrane conductances. In addition, since the basolateral HC membrane is enveloped by the calyx, the chord conductances between corresponding points on the SS and INST I–V curves were used to estimate the shifts in the equilibrium potential driving the net current. As a first approximation, the measured equilibrium potential was then used to estimate changes in ion concentrations in the cleft based on the Nernst potential.

These results are the first in mammals that support the Chen conjecture (34) that the extensive apposition between a Type I HC and its calyx creates a restricted space within which potassium is elevated in a manner previously demonstrated for the squid giant axon (35). By such accumulation, the equilibrium potentials of cleft-facing HC conductances are depolarized to levels sufficient for calcium influx, vesicle fusion, and quantal release onto ligand-gated channels in the calyx. In the calyx, the shift in the equilibrium potential is expected to depolarize the afferent to potentials close to the threshold for action potential generation. We assume that in mouse, as in turtle, this slow form of communication is bidirectional, and that [K^+^]_cleft_ can be elevated by potassium flux from either the HC or the calyx. In principle, this would open the possibility that in complex calyx-only endings, with multiple HCs enveloped by single or multiple calyces, the individual HCs may have unique constellations of channel expression whose differences is response dynamics are coupled through their common afferent.

### Prior recordings from solitary HCs

Patch recordings (36) from solitary auditory (37–40) and vestibular (41–44) HCs have revealed key physiological and biophysical mechanisms underlying their responses to stimulation. In the auditory system, essential features of frequency selectivity and tuning are often preserved after the cells are isolated (37, 40). Similar experiments in vestibular HCs, particularly Type I HCs, revealed conductances that begin to activate at potentials far more hyperpolarized than those necessary to activate typical calcium conductances and initiate quantal transmission (43). This apparent paradox—a large conductance that is fully activated at the nominal resting potential—would effectively short-circuit transduction currents through mechanoelectrical transduction channels and prevent the Type I HC from depolarizing significantly. This is not the case for Type II HCs, whose impedances near their resting potential resemble those of auditory HCs (43). Prior single-electrode recordings from vestibular HCs *ex vivo* (13, 16, 24) empirically evaluated Chen’s conjecture (34) and the related speculation that the large Type I HC potassium conductance is sufficient to elevate [K^+^]_cleft_, which depolarizes the HC by shifting E_K_, culminating in calcium influx (45) and synaptic transmission.

In the single-electrode recordings reported previously (16), the HC tail current apparent during repolarization “collapsed,” reflecting the depolarization of E_K_ with the expected reversal to an inward current upon return to the holding potential. Consequently, the elements of the fast component, including back-propagation of the action potential into the HC response and the rapid decrease in the inward calyx current induced by HC depolarization, were not examined in detail. This part of the study was limited to demonstrating that rapid transmission occurs near the assumed resting potential of both HC and calyx, but not at hyperpolarized potential where a majority of their voltage-sensitive conductances are closed [(8), Figure 10]. Instead, much of the initial effort in the dual-electrode recordings reported here focused on potassium ion accumulation underlying the slow component of bi-directional transmission.

### Identity of underlying potassium conductances remains unresolved

Based on prior work (8, 13, 14, 16, 24), including the demonstration that vestibular HCs express a wide variety of potassium channels (46), it is not surprising that HC potassium conductances are implicated as a major contributor to HC-calyx signaling interactions. These initial data in mouse suggest that two HC conductances may be involved in elevating [K^+^]_cleft_, although this is far from certain. With HC depolarization, the net outward ionic currents were best fit using the sum of two exponential recovery functions. In principle, this could result from the responses of two conductances with simple kinetics or a single conductance with complex transitions between open, closed, and inactivated states. The problem is further complicated by the fact that the HC currents evoked during step depolarizations show increases in the face of an elevation in the potassium equilibrium potential, a fact that would be expected to progressively decrease the driving force on the conductances during the step. As a result, the macroscopic current is not a simple reflection of the kinetics of the underlying potassium conductance(s).

Prior pharmacological studies of the potassium conductances in Type I HCs have often used external 4-AP to block the conductance underlying I_K(LV)_, a current that activates at hyperpolarized potentials. In the present experiments, we exploited the fact that with intracellular solutions at pH 7.2, membrane permeant 4-AP is converted to the membrane impermeant form 4-AP^+^, presumably exerting its blocking effect primarily on I_K(LV)_ (8). This allowed us to isolate the postsynaptic effects attributable to the recorded cell. One potential caveat of this approach is the possibility that calcium-activated potassium channels (8, 47) were also blocked in a voltage- and concentration-dependent manner (48). We did not rule this out, since it would have required examining the dose–response curve of the current at multiple potentials, an endeavor requiring a herculean effort. Thus, we are reluctant to speculate at present about the taxonomy of potassium conductances that potentially contribute to the slow accumulation and elevation of [K^+^]_cleft_. It is also unclear whether the results of the pharmacological experiments using 4-AP^+^ alone or in combination with TEA reflect a progressive block of a single conductance or exert effects on two pharmacologically distinct conductances. By extension, if two distinct conductances do in fact exist, we have no evidence yet to determine whether they correlate with the two kinetically distinct components seen with HC depolarization.

### Evolutionary pressures for fast bidirectional coupling

Selective pressures to boost the fidelity and speed of vestibular sensory transmission have been exerted over the course of vertebrate evolution from aquatic, through terrestrial, to arboreal environments. This is largely the result of increased mobility of the head and neck on the torso, and the body on the appendicular skeleton. With occupation of the arboreal canopy and brachiation by our primate ancestors, an inability to respond faithfully and accurately to imposed, unexpected perturbations to programed motor acts could prove catastrophic. This is also true for birds, in which flight necessarily occurs in an environment where unexpected air turbulence can interfere with the ability to catch prey, avoid predation and obstacles, and land safely; negative outcomes that would compromise evolutionary/reproductive success. The functional requirement for rapid feedback to stabilize the eyes while hunting reaches its zenith in raptors such as the peregrine falcon, which can attain speeds of 200 mph while diving (49).

Evidence supporting adaptation of the vestibular sensors to these evolutionary pressures has been mounting. A clear demonstration at the level of cellular physiology was provided by single-electrode patch recordings of HC and calyx responses to stimulation of the transduction apparatus across frequencies (11). This notion was further supported by a demonstration that irregular vestibular afferents remain phase-locked at stimulus frequencies greater than those of auditory afferents (50). Further experimental and theoretical work was consistent with fast transmission evoked by sound and vibration stimuli in mammals (10). The dual-electrode recordings from mouse shown in the present study are in general agreement with these prior conclusions and that of recent theoretical modeling (9, 51).

### Resistive coupling

We describe the fast bi-directional electrical communication between the Type I HC and its enveloping calyx as resistive coupling. In doing so, we seek to distinguish this from other forms of intercellular signaling that have been described in the vestibular periphery [for review, see (12)]. Chemical synapses are defined by specific structural and functional attributes, including pre- and post-synaptic membrane proteins, and synaptic vesicles containing neurotransmitter molecules. A slower mode of communication, which is demonstrated in the present study, reflects potassium ion accumulation in the synaptic cleft subsequent to HC depolarization. Electrical signaling, instead, involves the transfer of information between adjacent excitable cells. Examples of this general category of intercellular communication include gap junctions, ephaptic transmission and the resistive coupling described in the present study. Gap junctions display distinct morphology and are not present in association with vestibular HCs (29). Ephaptic transmission was originally described as the exchange of electrical activity between adjacent axons (52, 53), and is currently used primarily to describe coupling between cortical neurons (54, 55) or cerebellar Purkinje cells (56) that evince temporal and biophysical properties that differ markedly from the electrical transmission demonstrated in the present study.

Naming of this interaction between HC and calyx as *resistive* is intended to acknowledge the central role played by the resistance of the synaptic cleft. As an order of magnitude, our results indicate that R_cleft_ = 10 MΩ and G_cleft_ = 100 nS. This means that the conductance to the perilymph through the cleft is of the same order of magnitude as the maximum HC and calyx conductances measured here in mouse and previously in turtle (8). According to Kirchhoff’s Current Law (57), the sum of the currents in our lumped circuit model at Figure 5B, point ‘c’ must equal zero. Therefore, any transduction current flowing into the apex of the HC and out the basolateral membrane (Figure 5B, ‘b’) must either flow out the cleft from γ to δ, or through the inner face of the calyx (Figure 5B, ‘d’).

Initially, when the inner face calyx conductance is small, the majority of the HC current flows out the cleft conductance. As the calyx depolarizes, more of the inner face conductances open, diverting a larger percentage of the current into it. As the depolarization of the HC is prolonged, elevation of the [K^+^]_cleft_ occurs, opening additional inner face channels and diverting more depolarizing current into the calyx. By this means, the cleft-facing conductances in the HC and calyx increase, and so does the coupling between them. The resulting current divides according to Kirchhoff’s Current Law along each limb of the HC, calyx, cleft circuit, as determined by Ohm’s Law. This culminates in bi-directional coupling that permits and facilitates fast orthograde and retrograde transmission between the HC and calyx. This is visible in the retrograde transmission of action currents and the orthograde sudden decrease in the inward calyx current upon repolarization of the HC (Figure 5A).

The pivotal position of cleft resistance can be illustrated by considering the extremes as example cases. At one extreme, if R_cleft_ = 0 Ω and therefore G_cleft_ = ∞ nS, there would be no interaction between the HC and calyx, since currents flowing in or out of either encounter zero impedance to flowing via the synaptic cleft to ground, but a measurable and often significant impedance to flowing into their synaptic partner. At the other extreme, where R_cleft_ = ∞ Ω, and G_cleft_ = 0 nS, if the HC and calyx conductances were aligned, one recreates the conditions of a gap junction: currents from each cell would flow freely into the other and none would flow via the synaptic cleft to ground. In practice, resistive coupling is accomplished without gap junctions, via cleft-facing conductances in HC and calyx balanced against what, we assume, is the fixed conductance of the synaptic cleft.

## Data availability statement

The original contributions presented in the study are publicly available. This data can be found here: https://indigo.uic.edu with DOI: 10.25417/uic.26319145.

## Ethics statement

The animal protocol under which the studies were performed, was reviewed and approved by UIC Institutional Animal Care and Use Committee (IACUC) in accordance with the Animal Care Policies of the University of Illinois Chicago. This institution has Animal Welfare Assurance Number D16-00290 (A3460.01) on file with the Office of Laboratory Animal Welfare (OLAW), NIH.

## Author contributions

DC: Conceptualization, Data curation, Funding acquisition, Investigation, Methodology, Project administration, Resources, Validation, Visualization, Writing – original draft, Writing – review & editing. GRH: Conceptualization, Supervision, Visualization, Writing – original draft, Writing – review & editing. JJA: Conceptualization, Data curation, Formal analysis, Funding acquisition, Investigation, Methodology, Resources, Software, Supervision, Validation, Visualization, Writing – original draft, Writing – review & editing.
